# Investigation of Suffocation Mechanisms in the Infant Sleep Environment Using a Mechanical Breathing Model Simulation

**DOI:** 10.7759/cureus.78852

**Published:** 2025-02-11

**Authors:** Ruth Barker, Michael D Leshner

**Affiliations:** 1 Queensland Injury Surveillance Unit, Jamieson Trauma Institute, Brisbane, AUS; 2 Forensic Engineering, National Academy of Forensic Engineers, Atlanta, USA

**Keywords:** carbon dioxide, sids, suffocation, suid, work of breathing

## Abstract

Mattress firmness has long been recognized as a protective factor for sudden unexpected infant death (SUID) with mandatory infant mattress firmness regulations enforced by the US Consumer Product Safety Commission (CPSC) since August 2022. Recent research has revealed that two mechanisms are involved in SUID due to suffocation: airflow resistance (AR) and carbon dioxide rebreathing (CO_2_RB). By gaining an in-depth understanding of these mechanisms, existing bedding products can be evaluated and new products designed with infant safety as the primary consideration. This paper describes experiments and their results using a mechanical breathing model to elucidate the relationship and differences between these two mechanisms of suffocation.

The model was configured to measure the work of breathing (WOB) and carbon dioxide (CO_2_) rebreathing in the setting of increasing airflow resistance and increasing CO_2 _storage capacity, simulating but separating both potential respiratory hazards in infant sleep environments. The first series of experiments simulate increasing airflow resistance by using orifices of decreasing diameter. The second series simulates increasing the CO_2_ storage capacity of the bedding material by using an open-ended tube surrounded by an increasing thickness of soft (low-resistance), porous polyfill material. The third series of experiments were conducted to investigate both hazard mechanisms in combination. The results show that increasing airflow resistance and CO_2_ rebreathing are independent suffocation hazards, each with its own adverse effects that may occur independently or in concert, with cumulative effects.

## Introduction

A sleep surface that is soft and can conform to the anatomy of an infant’s face is known to be potentially hazardous to sleeping infants. Mandatory infant mattress firmness regulation has been enforced by the US Consumer Product Safety Commission (CPSC) since August 2022 [1]. However, firmness is not the only protective factor involved in infant sleep safety. Incline has recently been recognized as a hazard, as it promotes both premature prone rolling and positional asphyxia due to the “chin on chest” positioning of the infant. A detailed review of this issue was conducted following the deaths of more than 70 infants in the United States from inclined sleep products [2]. In September 2023, the CPSC banned infant sleep products with an incline greater than 10 degrees for infants five months or younger [3,4]. Lateral incline (as experienced in rocking cradles) is also a hazard, where infants can become trapped in the dependent side of the cradle [5]. In addition, surfaces that are not flat and cause flexion (such as hammocks and curved slings) or hyperextension of the infant’s neck/spine also cause respiratory compromise [6,7]. Therefore, current recommendations are that infants should sleep on a firm, flat, level (horizontal) surface.

A firm, flat, level mattress is protective for an infant placed supine because it protects their airway position and reduces the likelihood of positional asphyxia. When an infant rolls prone, a firm, flat mattress is also protective as it reduces the depth of face engagement and facilitates an infant’s ability to rotate their face away or push up to disengage from the sleep surface. Similarly, safer bedding options avoid the use of soft padded elements that can conform to an infant’s face such as pillows, quilts, bumpers, comforters, doonas, and snuggle “nests” with soft raised sides. Several of these products are subject to new US product safety rulings, with cot bumpers banned [8] and products such as “nests” and other previously unregulated “infant sleep products” now required to meet existing bassinet and cradle standards [9].

When infants are found lifeless in an unnatural position but with their faces unencumbered, positional asphyxia may be the cause of death, and the infant’s position is circumstantial evidence. Positional asphyxia is an extreme form of airway resistance and includes neck hyperflexion/extension (increasing airway resistance) and anterior curled positions (causing abdominal and chest wall movement restriction).

When an infant’s face engages, even partially, with a sleep surface or bedding, airflow to and from the infant can potentially be restricted, and/or the material can store the exhaled “stale air.” When this happens, the infant needs to work harder to breathe and is potentially breathing air that has lower oxygen (O_2_) and higher carbon dioxide (CO_2_) levels than fresh air. This is more likely to occur if the sleep surface or bedding is thick, flexible, or soft [10].

When infants are found lifeless with their faces engaged with the sleep surface or bedding, the medical examiner often diagnoses “suffocation” as the cause of death. However, little or no evidence may exist to determine whether death was caused by suffocation due to airflow resistance (AR) or suffocation due to carbon dioxide rebreathing (CO_2_RB) or both. Although this distinction may seem moot after the fact, a clear understanding and measurement of sleep surface and bedding performance with respect to each of these hazard mechanisms are important in mitigating suffocation hazards through design.

The purpose of this study is to simulate and quantify two different types of potential breathing hazards, individually and in combination, to help understand the multiple causes of suffocation.

## Materials and methods

Work of breathing (WOB) and airflow resistance

Work of breathing (WOB) is the amount of energy per unit of time needed by the respiratory muscles to produce enough ventilation and respiration to meet the metabolic demands of the body. WOB is the product of pressure and volume integrated over each breath [11]. During the inhalation portion of the breathing cycle, work is done by muscles to expand the lungs. When the muscles relax, the lungs passively deflate. This is only passive when there is no resistance to exhalation. If there is resistance (internal or external) to airflow, the muscles need to work harder to inflate and sometimes to deflate the lungs. In the case of partial airflow resistance, prolonged exposure can exhaust and overcome an infant. An infant’s respiratory system is further compromised by dynamic mechanical performance due to airway and chest wall compliance, as well as dynamic metabolic performance affected by gestational age, intercurrent illness, energy intake and stores, and genetic factors. In the case of extreme airflow resistance, an infant may not be able to work hard enough to overcome the resistance, with fatal consequences.

A mechanical breathing model was investigated at Leshner and Associates in the Baby Breathing Laboratory, Elkton, Maryland, in order to measure the work of breathing. The mechanical breathing model is adapted to measure the pressure and volume in the lungs as they change through the breathing cycle. Pressure and volume data are used to calculate the area on the negative side of the pressure-volume (p-v) diagram, which is a measure of the WOB in the model and a proxy for the total WOB in an infant.

Carbon dioxide rebreathing

When an infant’s face contacts a material that can store gas, such as soft bedding, some of the exhaled breath containing CO_2_ and depleted O_2_ may be stored within the bedding material and returned to the infant upon the next inhale, resulting in their rebreathing an elevated concentration of CO_2_ and decreased concentration of O_2_, relative to fresh air. Inhaled fresh air has a partial pressure of oxygen (pO_2_) of 160 mm Hg and a partial pressure of carbon dioxide (pCO_2_) of 0.3 mm Hg. Exhaled air in contrast has a pO_2_ of 116 mm Hg and a pCO_2_ of 35 mm Hg. Medical monitors measure CO_2_ and display the partial pressure as end-tidal CO_2_ or etCO_2_, measured in mm Hg (Table 1).

**Table 1:** Oxygen and carbon dioxide concentration and partial pressure in fresh air and exhaled breath

	Partial pressure	Concentration
	(mm Hg)	(vol%)
Oxygen in fresh air	160	21
Oxygen in exhaled breath	116	16.4
Carbon dioxide in fresh air	0.03	0.04
Carbon dioxide in exhaled breath	35	4.4

Rebreathing exhaled breath is known to be a potential hazard for infants and is believed to be a factor in sudden unintended infant death (SUID). CO_2_ rebreathing (CO_2_RB) and oxygen depletion are forms of suffocation, and death can occur without significant resistance to airflow [12,13].

Mechanical breathing model

Studies of this type cannot practically be performed on live infants, so mechanical breathing models have been developed and described in which CO_2_ gas is metered into an externally actuated “lung” breathing through a surrogate infant probe that has been designed to replicate the midface and nares of an infant [14-16]. In this study, the probe was replaced by orifices of decreasing size and by adding layers of porous material to create gas storage capacity (GSC). The resulting concentrations of CO_2_ and O_2_ in the model’s lungs were allowed to change and reach an equilibrium level and were measured using a CO_2_ analyzer (Horiba VIA 510, Kyoto, Japan) and an O_2_ analyzer (Thermox A/S II Oxygen Analyzer, Ametek Process Instruments, Inc., Newark, DE). The model does not include any of the biofidelic features such as variable lung volume, compliance, or airway size nor the physiological compensatory mechanisms of an infant, such as increases in respiratory rate, pulse, or tidal volume. In particular, the model is unable to capture the dynamic factors that might further affect an infant’s respiration, particularly those associated with intercurrent illness and metabolic states such as fasting and airway secretions. The breathing model is designed as a test method and not an actual biofidelic representation. Accordingly, the model may both over- and underestimate the elevation of CO_2_ and the depletion of O_2_, compared with the compensated response of a live infant to the same external stressors.

The breathing model used in this study makes use of twin elastic bellows having a compliance of 0.7 mL/mbar, similar to an infant’s lungs. A laser distance sensor (Micro-Epsilon opto-NCDT, Ortenburg, Germany) monitors the free end of one lung to monitor its position, which indicates changes in lung volume. The lungs are actuated by an external vacuum pump and control valves to intermittently supply suction or vent to the atmosphere in the space surrounding the lungs, as shown in Video 1. The lungs reciprocate between volumes of 30 mL and 65 mL for a tidal volume of 35 mL. With a breathing rate of 44 breaths per minute, the model simulates a sleeping infant. The selection of lung volumes and breathing rate are consistent with the study performed by Carleton et al. [14] and used in the author’s prior studies [15,16].

**Video 1:** Soft lungs breathing at infant volume and breathing rate By using two linear bellows, the lung volume can be measured dynamically and volume calculated from its linear displacement

Experimental apparatus and methodology

The experimental apparatus for the measurement of CO_2_RB is described in the author’s prior publications, with the exception of the lungs. Rather than a mechanical piston-cylinder pump that produces sinusoidal motion in the lung, a pair of soft lungs was employed to better simulate the motion of the lungs in response to pressure differences and allow the measurement of the volume as it changes. The soft lungs produce a more biofidelic pressure waveform than a mechanical pump. The motion of the lungs is programmed to move a fixed volume of air at a fixed frequency, unlike a live infant that adjusts its respiratory rate and tidal volume to control pCO_2_ and pO_2_. Where an infant may underinflate in the face of increasing airflow resistance and dynamic lung and airway factors, the breathing model has a fixed tidal volume, though this requires rising negative pressure to generate. Video 1 shows the soft lungs breathing.

In each test series, rebreathing measurements were repeated three times, with the average reported. For pressure and volume data, 10 cycles were recorded, with work calculated for a single cycle in a series of identical cycles. Video 2 illustrates the test methodology, with CO_2_ measured in the lungs and end-tidal CO_2_ measured breath by breath at the probe.

**Video 2:** Demonstration of rebreathing measurements End-tidal CO_2_ (Medtronic Capnostream 35, Minneapolis, MN) and volumetric CO_2_ measured at the lungs (breathmeter.com) CO_2_: carbon dioxide

Pressure and volume measurement

**Figure 1:**
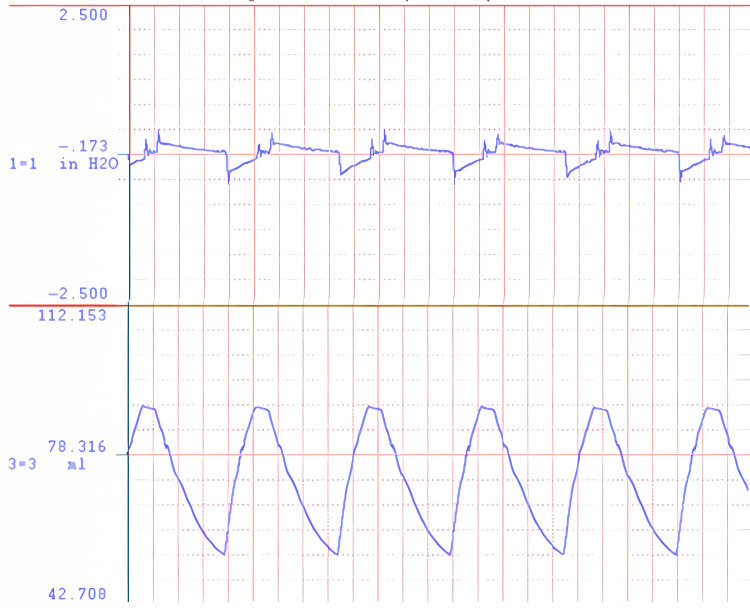
Pressure and volume waveforms for flow through an open-ended tube

**Figure 2:**
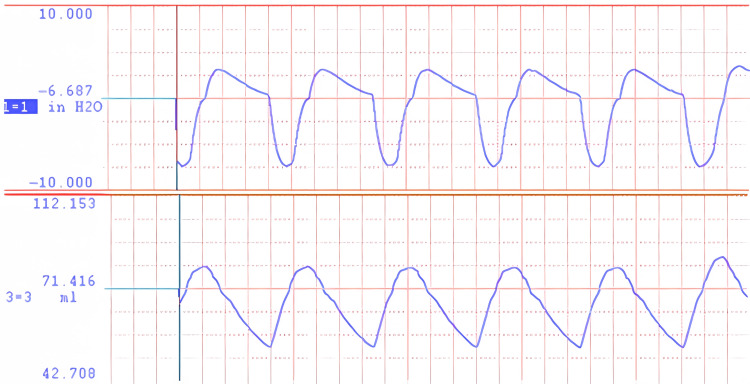
Pressure and volume waveforms for flow through a 1.07 mm orifice

Pressure in the breathing circuit was measured using a fast-response, low-range pressure transducer (Validyne P17, Northridge, CA). The laser distance measurement was calibrated against total lung volume. Pressure and volume data were recorded using a DATAQ DI-245 (Akron, OH) for signal acquisition and WINDAQ software (DATAQ, Akron, OH) for display and recording. Two examples of pressure and volume records are shown in Figure 1 and Figure 2 representing the extremes in airflow resistance. Figure 1 shows the pressure and volume data for breathing through an open-ended tube 300 mm in length and 5 mm in internal diameter. Even this large passage causes some airflow resistance. Figure 2 shows the pressure and volume data for breathing through the same tube with an orifice of 1.07 mm in diameter attached. The time scale is 44 breaths per minute.

First test series: Effect of increasing airflow resistance without increasing gas storage

**Figure 3:**
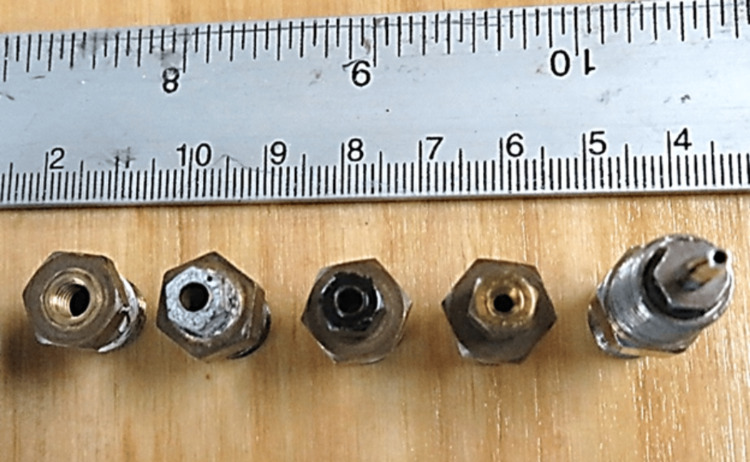
Orifices arranged in decreasing internal diameter

The first series of tests were performed sequentially with the 300 mm-long open-ended tube and then the same tube connected to increasingly restrictive orifices with an internal diameter of 4.03, 3.25, 2.64, 1.93, and 1.07 mm as shown in Figure 3.

Second test series: Effect of increasing gas storage

A second series of tests were performed by surrounding the open-ended tube as described above with increasing thicknesses of low-density polyfill material. This series was designed to utilize a storage medium that would promote the rebreathing of exhaled air while offering very little airflow resistance. The polyfill material was applied in an increasing number of layers, initially over, then sandwiching (over and under), and finally circumferentially wrapping around the tube opening, with each addition increasing the CO_2_ storage capacity at the tube opening.

Third test series: Effect of increasing airflow resistance and gas storage capacity in combination

Three different levels of airflow resistance were evaluated with three different levels of gas storage capacity to yield nine combinations, as shown in Table 2.

**Table 2:** Combinations of gas storage capacity and airflow resistance selected for the third series of tests

Gas storage	Airflow resistance
2 layers of polyfill	Open tube
4 layers of polyfill	3.25 mm orifice
Wrapped in polyfill	1.93 mm orifice

## Results

Relationship between work, CO_2_, and O_2_ concentration

**Figure 4:**
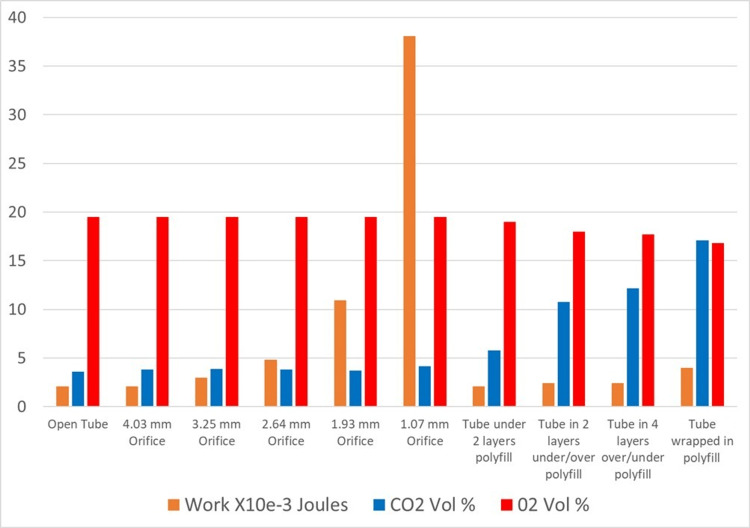
Relationship between work, CO2 rebreathing, and oxygen concentration in the lungs CO_2_: carbon dioxide

Figure 4 shows the measured work in Joules, with measured carbon dioxide and oxygen concentrations expressed as a volume percent for the first and second test series. Increasing airflow resistance increases the work of breathing but has no effect on O_2_ or CO_2_ levels in the mechanical model. However, increasing the gas storage medium results in a dramatic rise in the CO_2_ level and a smaller corresponding fall in the O_2_ level.

WOB calculation

**Figure 5:**
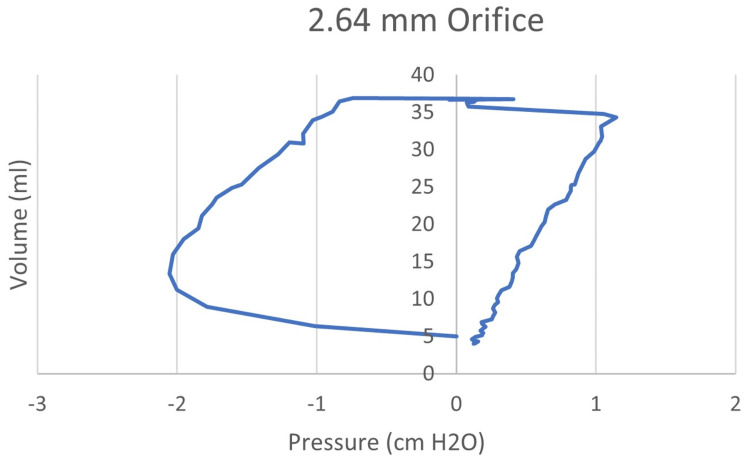
Pressure-volume diagram for one breathing cycle of flow through a 2.64 mm orifice The area on the negative side of the pressure-volume diagram represents the work of breathing

To determine the work of breathing, the area within the pressure/volume diagram is calculated. The area on the negative pressure side of the diagram represents work performed by the infant model. Work per cycle is multiplied by the breathing rate to calculate the total work per minute. An example of the p-v diagram is shown in Figure 5 with the area under the curve on the negative side of the pressure scale representing work per breath.

First test series: Airflow resistance without increasing gas storage

Pressure and volume data were recorded, and the work of breathing was calculated for each test condition. Orifices of decreasing diameter were used to increase resistance to airflow without increasing gas storage capacity. As the flow path becomes narrower with increasing resistance, the work increases dramatically, while the CO_2_ rebreathing remains constant.

**Figure 6:**
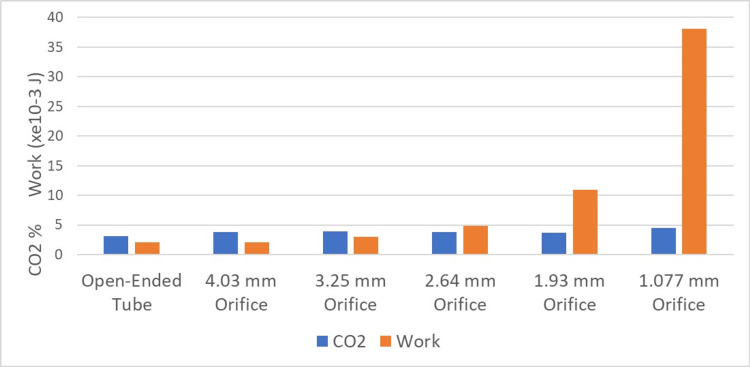
CO2 level and work: increasingly restrictive orifices without increasing gas storage medium CO_2_: carbon dioxide

As shown in Figure 6, increasing airflow resistance creates a significant increase in work without a change in CO_2 _percent, measured in the lungs.

Second test series: Increasing gas storage with minimal increase in airflow resistance

**Figure 7:**
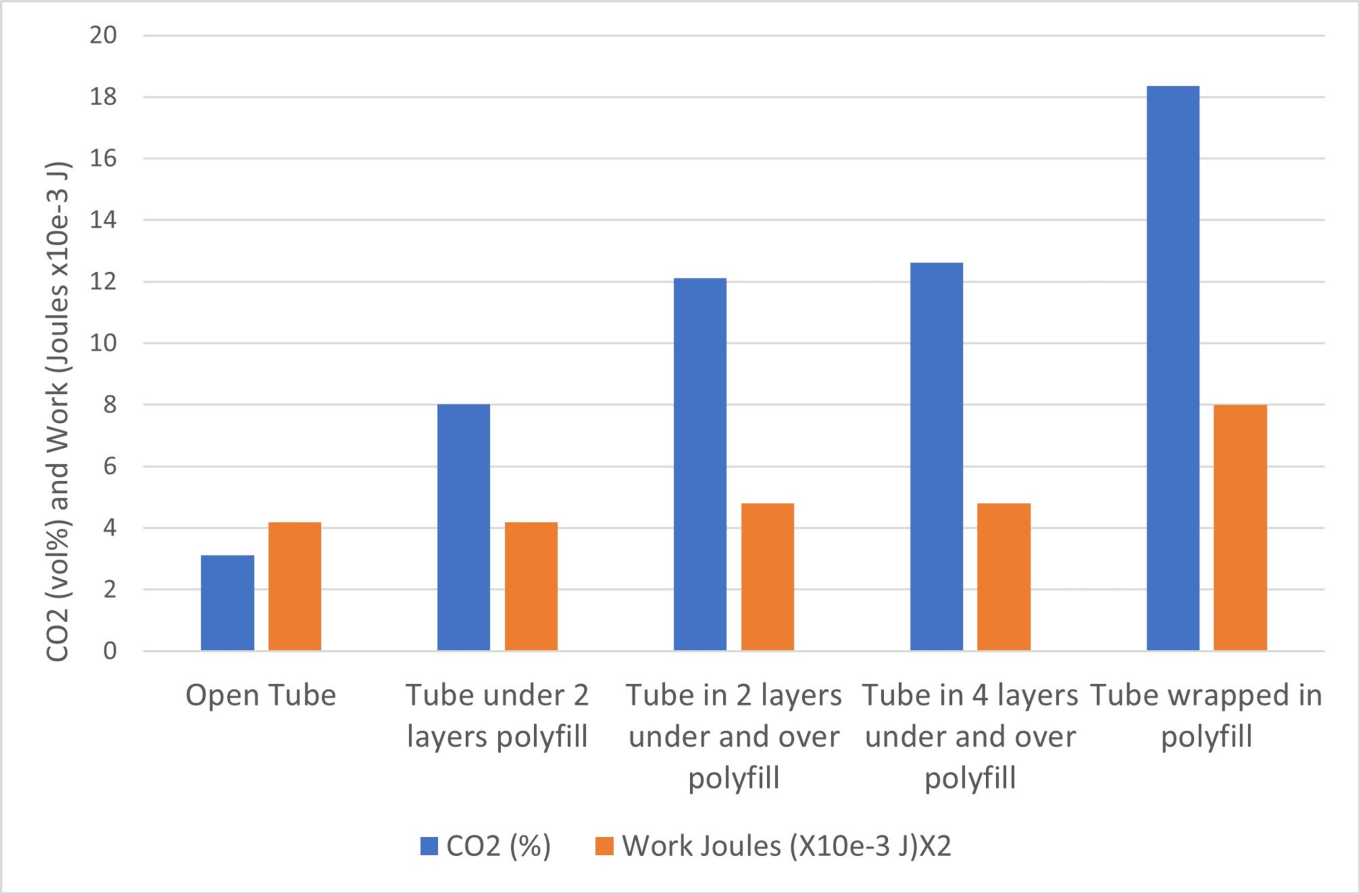
Carbon dioxide (CO2) rebreathing and work of breathing with increasing gas storage capacity

The second test series, shown in Figure 7, was designed to replicate the CO_2_ rebreathing hazard by using increasing layers of polyfill material, known to store gas within its fibers, promoting rebreathing as illustrated in Figure 4.

The second test series resulted in a significant increase in CO_2_ measured at the lungs as layers of material were added, with only a slight increase in WOB, as shown in Figure 7.

Third series: Effect of increasing airflow resistance and gas storage capacity in combination

Three resistance conditions (open-ended tube, 3.25 mm orifice, and 1.93 mm orifice) were combined with three storage conditions (two layers, four layers, and eight layers).

**Figure 8:**
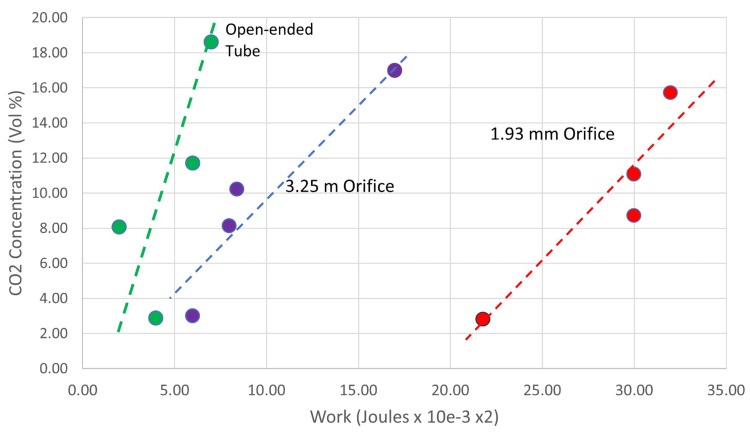
Carbon dioxide (CO2) rebreathing and work of breathing for three levels of airflow resistance and increasing gas storage media

As shown in Figure 8, both the WOB and CO_2_ level increase together when increasing airflow resistance and CO_2_ storage are applied in combination.

## Discussion

This series of experiments demonstrates the effects of independently increasing airflow Resistance (AR) and gas storage capacity (GSC) on CO_2_RB and WOB in an infant breathing model. The series was designed to replicate factors that might be experienced by a prone infant whose face has become engaged with the sleep surface, though, unlike a real infant, the model lungs do not tire nor respond to physiological cues such as increases in pCO_2_ and AR. A limitation of this study is that CO_2 _concentrations measured in the model may overestimate or underestimate CO_2_ concentrations measured in a live infant.

In real life, an infant lying on their back on a firm level surface will have an unimpeded inflow of fresh air and an expiration of stale air with an etCO_2_ of approximately 35 mm Hg (corresponding to a volumetric CO_2_ concentration of approximately 4%).

When an infant rolls prone and their face engages with a sleep surface, AR will determine whether they are able to breathe into and out of that surface or are required to turn their face. Ensuring a firm, flat, level surface mitigates the risk of the infant’s face engaging completely with the surface and increases the infant’s chances of self-rescue if they encounter excessive AR, by the infant lifting or turning their head/face from the surface [5-7].

Where the sleep surface is air permeable and the infant is able to overcome the AR, the GSC will determine what mix of expired air an infant might inhale. Some sleep surfaces are highly air permeable and yet have variable gas storage capacities depending on their ability to dissipate expired gases. Therefore, dependent on the properties of the sleep surface, an infant, with their face wholly or semi-engaged, will experience a variable degree of AR and will breathe a variable mixture of expired air from the sleep surface together with fresh entrained air.

The first test series modelled independent increases in AR by decreasing orifice sizes in the breathing apparatus. Increasing AR significantly increased WOB with no effect on CO_2_ concentration. With no gas storage medium to store exhaled breath, there could be no CO_2_RB (Figure 6). However, in real life, increasing WOB will increase CO_2_ generation as the infant’s muscles work harder. Increasing AR can arise due to factors that are both intrinsic and extrinsic to the infant. While this paper focuses on sleep surface factors (extrinsic), in real life, intrinsic factors (such as neck flexion/hyperextension, airway anatomy/compression, or tracheal/nasal secretions) can increase AR and further exacerbate infant WOB requirements and CO_2_ generation.

The second test series models the effect of independently increasing GSC by using an open-ended tube with added layers of polyfill material as a storage medium for exhaled gas. This test was designed to reproduce rebreathing from the sleep surface in the absence of significantly increased AR. As expected, adding layers of material had a strong effect on CO_2_RB with only a marginal effect on WOB (Figure 7). This suggests that suffocation by CO_2_RB due to the storage of exhaled gas within bedding material may also be an independent infant sleep hazard.

A prone infant, whose face remains engaged with and who continues to breathe into and out of a sleep surface, will simultaneously be exposed to both increased AR and CO_2_RB in a manner determined by the material and construction of the sleep surface. This is illustrated in Figure 8, which shows the relationship between the two independent hazard mechanisms. While, in the model, the effects of simultaneously increasing GSC and AR are summative, in the infant, the effects are likely compounding.

As an infant works harder against increased AR, more work generates more CO_2_. At the same time, any exhaled CO_2_ rebreathed by the infant from the sleep surface further exacerbates CO_2_RB. An infant responds to rising CO_2_ levels by increasing their respiratory rate and tidal volume in an attempt to normalize the CO_2_. This physiological response again requires an increase in WOB generating more CO_2_.

This becomes a vicious cycle as the harder the infant works to take a breath, the more CO_2_ they generate, and the more CO_2_ they generate and rebreathe, the more they have to clear.

For infants, factors that increase respiratory effort risk overwhelming their energy reserves and physical limitations. As the infant tires, CO_2_ levels rise precipitously, causing CO_2_ narcosis, a rise in intracranial pressure due to vasodilatation, and eventually death.

Whether this occurs acutely or over a longer time period depends upon the severity of the insult (degree of AR and CO_2_RB), as well as infant factors such as gestational age, physical and metabolic status, and dynamic issues such as intercurrent illness, exposure to cigarette smoke/drugs and alcohol, or other environmental factors.

Factors that predominantly increase CO_2_RB with little effect on AR may have more subtle, slow-onset, and cumulative effects. For normal infants, increased respiratory effort is driven by rising CO_2_ levels, with CO_2_ homeostasis maintained through the fine-tuning of tidal volume and respiratory rate. However, several innate and dynamic factors may interfere with this homeostasis, and many of these factors are recognized as independent SUID risk factors: prematurity; low birth weight; exposure to cigarette, vape, or environmental smoke; intercurrent illnesses; and genetic/metabolic factors that affect acid-base regulation. An infant’s respiratory response to hypercapnia is variable, depending on gestational and post-gestational age, as well as context [17]. Medium to long-term exposure to elevated CO_2_ levels can alter the hypercapnia threshold and affect breathing patterns [18]. Many premature infants have a heightened tolerance for elevated CO_2_ levels [19]. Prone positioning can further diminish an infant’s respiratory response [20]. Exposure to elevated levels of CO_2_ can impact immune modulation, with negative effects in the context of infection and positive effects in regulating uncontrolled inflammation [17]. In addition, it may have long-term neurological impacts [21].

Counterintuitively, sleep surfaces, provided that they are firm, flat, and level and do not conform to the infant’s face, that have very high air impermeability (extreme AR) are the easiest for infants to breathe on. A small rotation of the infant’s face allows the inhalation of fresh air with no rebreathing from the sleep surface. The American Academy of Pediatrics (AAP) recommends the following: infants should be placed on a firm sleep surface (e.g., a mattress in a safety-approved crib) covered by a fitted sheet with no other bedding or soft objects to reduce the risk of sudden infant death syndrome (SIDS) and suffocation [9]. A vinyl-covered mattress and sheet are considered safe by the AAP, if it is sufficiently firm and flat, and have been associated with a reduced risk of sudden infant death syndrome (SIDS) in a large UK case-control study [22].

Alternatively, there are designs on the market that allow prone infants to breathe freely through sleep surfaces that are both highly air permeable (low AR) and have no GSC. They are capable of rapidly dissipating exhaled CO_2_ between breaths, ensuring minimal CO_2_RB. Such mattresses are higher cost and not available to the majority of families.

Although mattress firmness requirements have been mandated by the US CPSC since August 2022, at present, the risk of CO_2_RB posed by infant sleep surfaces is untested and unregulated. There are various claims of the “breathability” of sleep products without the term being adequately defined nor the products being tested to an agreed testing standard.

Recommendations

Infant respiratory hazards are often subtle, invisible, and difficult to recognize and detect. When the worst occurs, there is often no evidence to determine the exact cause of death.

“Back to Sleep” educational programs have been effective in reducing SUID associated with prone and side positioning [23,24]. However, infants who roll prone are still exposed to the independent but compounding hazards of AR and CO_2_RB when their sleep surface is too soft, is capable of storing CO_2_, and is sufficiently air permeable to allow the infant to rebreathe this stored CO_2_. There are currently industry standard test methods being developed both in the United States and Australia to measure the CO_2_ permeability of sleep surfaces. CO_2_RB for the prone infant can be minimized by designing sleep surfaces that, in addition to meeting firmness and configuration requirements (are both flat and level), do not store hazardous amounts of CO_2_ either because they are largely air impermeable or are highly air permeable but allow for the rapid dissipation of CO_2_ between breaths.

## Conclusions

This study investigated two potential infant breathing hazards, separately and in combination. Mechanical resistance to airflow causes the infant to work harder to maintain homeostasis, generating more CO_2_. Carbon dioxide rebreathing increases with greater gas storage capacity within the bedding and causes an infant to adjust its breathing rate and tidal volume in order to clear more CO_2_. It was found that these two hazard mechanisms are independent and can occur simultaneously. The combined effect of both hazards is likely compounding in real-life settings.
